# Supplementary material for: Identification of photoperiod-induced specific miRNAs in the adrenal glands of Sunite sheep (Ovis aries)
Source: Front Vet Sci. 2022 Jul 22;9:888207. doi: 10.3389/fvets.2022.888207 (PMC9354845; doi:10.3389/fvets.2022.888207)
Supplement: Supplementary file 1 [file Data_Sheet_1.ZIP › Supplementary file/miRDeep2 file.zip!/miRDeep2 file/Novel_Structure/LP42A2/Novel_7.pdf]

Provisional ID	:	NC_019476.2_29854
Score total	:	1
Score for star read(s)	:	-1.3
Score for read counts	:	0
Score for mfe	:	0.7
Score for randfold	:	1.6
Score for cons. seed	:	
Total read count	:	203
Mature read count	:	202
Loop read count	:	0
Star read count	:	1

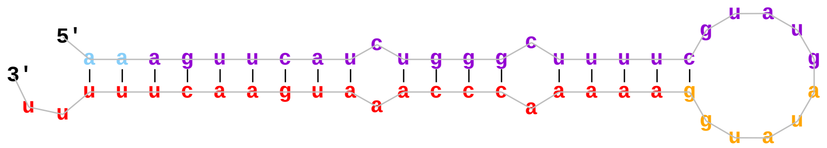

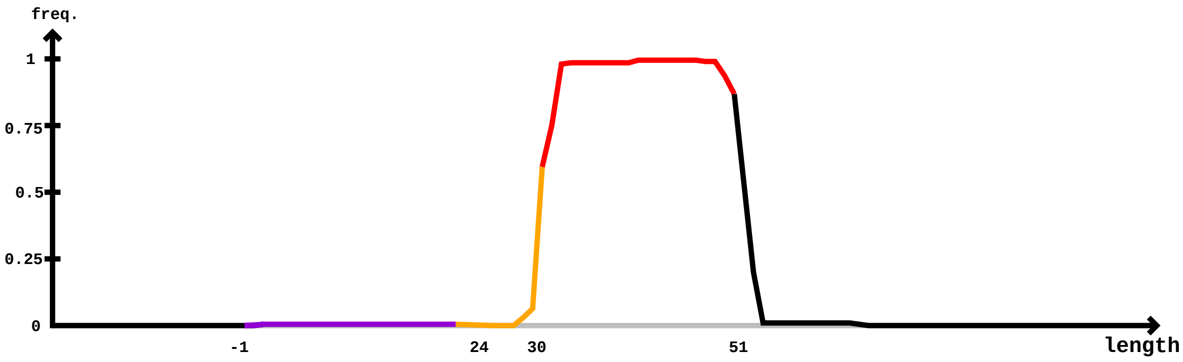

Star Mature

```
5'- uaauacuggauugaccaaaaaguucaucugggcuuuucguaugauauggaaaaacccaaaugaacuuuuugaccaacccaauaccuauuuguaugcuucuccugcucaguugc  -3'   obs
    uaauacuggauugaccaaaaaguucaucugggcuuuucguaugauauggaaaaacccaaaugaacuuuuugaccaacccaauaccuauuuguaugcuucuccugcucaguugc        exp
    ......(((.(((..(((((((((((.((((.(((((..........))))).)))).)))))))))))..))).)))((((......))))((.......))........   reads   mm   sample
    ....................aguucaucugggcuuuucguaug......................................................................   1       0    seq
    ...............................................ggaaaaacccGaaugaacuuu.............................................   1       1    seq
    ...............................................Ugaaaaacccaaaugaacuuu.............................................   2       1    seq
    ...............................................ggaGaaacccaaaugaacuuu.............................................   1       1    seq
    ...............................................Ugaaaaacccaaaugaacuuuu............................................   1       1    seq
    ...............................................ggaaaaacccaaaugaacuuuA............................................   1       1    seq
    ................................................gaaaaacccaGaugaacuuuu............................................   6       1    seq
    ................................................gaaaaacccaGaugaacuuuuu...........................................   1       1    seq
    .................................................aaaaacccaGaugaacu...............................................   1       1    seq
    .................................................aaaaacccaGaugaacuuu.............................................   7       1    seq
    .................................................aaaaacccGaaugaacuuuu............................................   2       1    seq
    .................................................aaaaacccaGaugaacuuuu............................................   4       1    seq
    .................................................Caaaacccaaaugaacuuuuu...........................................   1       1    seq
    .................................................aaaaGcccaaaugaacuuuuu...........................................   2       1    seq
    .................................................aaaaCcccaaaugaacuuuuu...........................................   2       1    seq
    .................................................aaaaacccaaaugaacuuuuG...........................................   1       1    seq
    .................................................aaaaacUcaaaugaacuuuuu...........................................   1       1    seq
    .................................................aaaaacccaaaugaacuuuuA...........................................   2       1    seq
    .................................................aaaaacccaGaugaacuuuuu...........................................   42      1    seq
    .................................................aaaaacccaaaugaacuuuuuA..........................................   6       1    seq
    .................................................aaaaacccaaGugaacuuuuug..........................................   1       1    seq
    .................................................Caaaacccaaaugaacuuuuug..........................................   3       1    seq
    .................................................aaaaacccaaaugaacuuuuuU..........................................   2       1    seq
    .................................................aaaaacUcaaaugaacuuuuug..........................................   9       1    seq
    .................................................aaaaacccaGaugaacuuuuug..........................................   2       1    seq
    .................................................aaaaGcccaaaugaacuuuuug..........................................   6       1    seq
    .................................................aaaGacccaaaugaacuuuuuga.........................................   2       1    seq
    .................................................aaaaacccaaaugaacuuuuugU.........................................   5       1    seq
    .................................................aaaaacccaaaugaacuuuuuga.........................................   4       0    seq
    .................................................aaaCacccaaaugaacuuuuuga.........................................   1       1    seq
    .................................................aaaaGcccaaaugaacuuuuuga.........................................   3       1    seq
    ..................................................aaaacccGaaugaacuuuuu...........................................   1       1    seq
    ..................................................aaaacccaaaugaacuuuuug..........................................   12      0    seq
```

Star Mature

```
uaauacuggauugaccaaaaaguucaucugggcuuuucguaugauauggaaaaacccaaaugaacuuuuugaccaacccaauaccuauuuguaugcuucuccugcucaguugc
..................................................aaaacccaaGugaacuuuuug..........................................    1    1    seq
..................................................aaaacUcaaaugaacuuuuug..........................................    6    1    seq
..................................................aaaacccaaaugaacuuuuuU..........................................    1    1    seq
..................................................aCaacccaaaugaacuuuuug..........................................    1    1    seq
..................................................aaaacccaaaugaacuuuuuA..........................................    4    1    seq
..................................................aaaacccaaaugaacuuuuugU.........................................    4    1    seq
..................................................aaGacccaaaugaacuuuuuga.........................................    2    1    seq
...................................................aaacccGaaugaacuuuuu...........................................    28   1    seq
...................................................aaacccaaaugaacuuuuug..........................................    2    0    seq
...................................................aaacccaaaugaacuuuuugU.........................................    1    1    seq
...................................................aaacccGaaugaacuuuuuga.........................................    16   1    seq
....................................................aacccaaaugaacuuuuuga.........................................    1    0    seq
...........................................................augaacuuuuugaccaacccaaA...............................    1    1    seq
...........................................................augaacuuuuugaccaacccaaAa..............................    1    1    seq
```
